# Experience and coping strategies of parents of children with autism: A qualitative study

**DOI:** 10.1371/journal.pone.0339349

**Published:** 2025-12-23

**Authors:** Archana Bagale, Anil Raj Ojha, Machhindra Lamichhane

**Affiliations:** 1 School of Nursing and Midwifery, Patan Academy of Health Sciences, Kathmandu, Nepal; 2 Department of Pediatrics, Patan Academy of Health Sciences, Kathmandu, Nepal; 3 Department of Pediatrics, Civil Service Hospital, Kathmandu, Nepal; Father Muller Charitable Institutions, INDIA

## Abstract

**Background:**

The neurodevelopmental disorder known as autism spectrum disorder (ASD) affects people of all racial, ethnic, and socioeconomic backgrounds. Although the study of autism is burgeoning with important implications both for public health and society, there is little research exploring the experiences of raising a child with autism spectrum disorder (ASD) from the Parent’s perspectives. The aim of this study was to explore experiences and coping strategies of parents of children with ASD in Nepal.

**Methodology:**

A descriptive phenomenological design explored the lived experiences of nine parents raising children with ASD in Nepal. Participants were purposively selected from the Pediatric OPD of Patan Academy of Health Sciences. Data were collected through in-depth, semi-structured interviews in Nepali and analyzed using Colaizzi’s method. The interview guide, adapted from Batchelor (2017) and the Chronic Sorrow Instrument, explored emotional, social, financial, and caregiving experiences. Researcher bias was minimized through bracketing and reflexive journaling, and trustworthiness ensured via member checking, audit trails, and peer debriefing. Ethical approval was obtained from the PAHS Ethical Review Board, with informed consent and confidentiality maintained.

**Results:**

Five major themes emerged: **psychological impact** (emotional burden, hope and uncertainty, guilt), **physical impact** (fatigue, sleep deprivation, safety concerns), **social impact** (isolation, stigma, family sacrifices), **career impact** (job loss, reduced opportunities), and **financial impact** (high costs, limited resources). Coping strategies included crying, listening to religious music (bhajans), meditation, and practicing positive thinking. Some parents reframed their experience as an opportunity to help others.

**Conclusion:**

Parents raising children with autism in Nepal face profound emotional strain, physical fatigue, social isolation, career disruption, and financial pressure. These challenges were especially evident among mothers, who formed the majority of caregivers. Despite early diagnosis and continued care, families reported limited resources and persistent social stigma. Yet, many parents showed determination and resilience in supporting their children’s development. These findings emphasize the need to strengthen family-centered support, accessible services, and community awareness to better address the lived realities of caregivers.

## Introduction

Autism Spectrum Disorder (ASD) is a neurodevelopmental disorder that affects individuals of all racial, ethnic, and socioeconomic backgrounds. ASD is a lifelong condition characterized by persistent impairments in social interaction, communication (both verbal and nonverbal), and the presence of restricted, repetitive patterns of behavior, interests, or activities [1]. The diagnostic criteria for ASD are outlined in the *Diagnostic and Statistical Manual of Mental Disorders, Fifth Edition (DSM-5)*. For an ASD diagnosis, children must demonstrate challenges in social communication and restricted, repetitive behaviors or interests, with symptoms typically appearing in early childhood. Some children may exhibit noticeable symptoms within the first 12 months of life, while others may not display clear signs until 24 months or later. Some children may temporarily lose skills they had previously acquired [1].

Children diagnosed with ASD often struggle to form lasting peer relationships and may face unique communication challenges. These challenges necessitate adjustments in educational curricula and individualized support to meet their specific needs [2]. Additionally, many children with ASD engage in self-stimulatory motor behaviors, such as rocking, hitting objects, biting themselves, or spinning [2].

Globally, Autism Spectrum Disorder (ASD) affects about 1 in 100 children, though prevalence varies across regions and studies [3]. For instance, a South Korean study reported a rate of 2.6% [4], while the U.S. Centers for Disease Control and Prevention (CDC) estimated that 1 in 54 children were identified with ASD by age 8 in 2020, 10% increase from 2014 [5]. In Asia, Shrestha et al. reported six cases per 1,000 individuals in Southeast Asia, with a higher prevalence among males [6]. Estimates further suggest there are approximately two million individuals with autism in India, 1.1 million in China, and 0.65 million in the UK [7].

Despite growing global awareness, Nepal lacks reliable prevalence data and in-depth qualitative research on families’ lived experiences. It is estimated that 250,000–300,000 individuals in Nepal may have autism, with 60,000–90,000 significantly affected [8]. However, limited diagnosis, awareness, and support services highlight the urgent need to explore the emotional, social, and financial challenges faced by parents raising children with ASD. This study seeks to fill that gap by exploring their lived experiences.

In the scientific community, the study is expected to broaden the global discourse on autism by incorporating perspectives from an underrepresented low- and middle-income country. Findings may inform culturally sensitive interventions, shape inclusive policies, and promote a shift toward strength-based and community-supported approaches to neurodiversity in Nepal and comparable South Asian contexts.

### Objective of the study

To explore the experiences and coping strategies of parents raising children with autism.

## Methods

### Design

A qualitative descriptive phenomenological research design was employed to explore the lived experiences of parents raising children with autism. Grounded in the philosophical tradition of Edmund Husserl, this approach seeks to understand human experiences as they are lived, without interpretation or theorization. The aim was to describe the essence of these experiences through the perspectives of those who have directly encountered the phenomenon.

This design emphasizes bracketing (epoché) the process by which researchers set aside their own assumptions, beliefs, and prior knowledge to engage with participants’ narratives as authentically and objectively as possible. By focusing on the description of the phenomenon in its pure form, the study aimed to uncover rich, in-depth insights into the psychological, social, physical, and financial realities faced by parents of children with autism in Nepal.

The descriptive phenomenological design is particularly suited for studies where little is known about the phenomenon and where the goal is to capture the meaning of lived experience in the words of those who experience it.

### Research setting

The study was conducted at the Pediatric Outpatient Department (OPD) of Patan Academy of Health Sciences (PAHS), Nepal.

### Sampling unit and sample size

The sampling unit consisted of parents (either father or mother) who were directly involved in the daily care and upbringing of a clinically diagnosed child with autism spectrum disorder (ASD). A purposive sampling strategy was employed to recruit participants who could provide rich, firsthand descriptions of their lived experiences.

A total of nine parents were selected from those attending the private pediatric outpatient clinic at Patan Hospital, Nepal. This sample size aligns with the methodological guidance for descriptive phenomenological research, which emphasizes depth over breadth and typically involves small sample sizes (usually fewer than ten participants) to enable an in-depth exploration of the phenomenon. Participants were chosen based on their ability to articulate and reflect upon their experiences, which is essential for capturing the essence of the lived phenomenon in phenomenological inquiry [9,10].

### Inclusion criteria and exclusion criteria

The study included parents who had at least one clinically diagnosed autistic child below 18 years of age and were willing to participate in the research. Parents who were unwilling to participate voluntarily or who had mental health issues or communication difficulties were excluded from the study.

#### The data collection instrument consisted of two main sections.

The study comprised two main parts. Part I focused on the sociodemographic profile of the parents and their children with autism, capturing essential background information such as age, education, occupation, and family composition. Part II consisted of open-ended questions aimed at exploring the parents’ lived experiences of raising a child with autism, providing insight into their emotional, social, and practical challenges, as well as the coping strategies they employed.

#### Participant recruitment and data collection.

Participants were recruited for this study from December **2024 to April 2025**. An interview guide was developed and adapted from Batchelor (2017), incorporating relevant elements from the Chronic Sorrow Instrument to capture the emotional dimensions of caregiving. In-depth, face-to-face interviews were conducted with parents who were directly involved in the dayto-day care of their child with autism. Each interview session lasted approximately 40–60 minutes, with two to three sessions per participant, conducted until data saturation was achieved. Interviews were digitally recorded using a Redmi smartphone and an audio recorder, and all recordings were securely stored in Google Drive. Field notes were also maintained to capture non-verbal cues and contextual information. All interviews were conducted in simple Nepali to ensure clarity and comfort for participants. The recordings were transcribed verbatim and translated into English, with translated transcripts reviewed by an expert to ensure accuracy, linguistic consistency, and preservation of meaning.

Interviews were conducted in simple Nepali to ensure clarity and avoid technical jargon. Both verbal and nonverbal cues were carefully observed and documented throughout the interactions. A digital recorder was pre-checked for functionality, and all interviews were transcribed verbatim. To minimize researcher bias, bracketing was practiced during data collection and analysis. The accuracy and credibility of the data were enhanced through repeated readings, cross-checking transcripts with audio recordings, and reconfirmation with participants. Consistency was maintained by conducting two to three interviews with each participant. To ensure confirmability, the findings were discussed with a validated research advisor. Finally, transferability was strengthened through the use of thick, contextual descriptions and purposive sampling to capture diverse parental perspectives.

Ethical approval for the study was obtained from the Ethical Review Board of the Patan Academy of Health Sciences (PAHS) (Ref. No.: PAHS-ERB-2024/097). All participants provided written informed consent prior to participation, including permission for audio recording, use of anonymized quotations, and inclusion of field notes. The study adhered to the ethical principles outlined in the 1964 Helsinki Declaration and its subsequent amendments. Confidentiality was maintained through anonymization of transcripts and de-identification of personal identifiers, with data securely stored in password-protected digital folders accessible only to the research team. Participation was entirely voluntary, and participants were fully informed of their right to withdraw at any stage without penalty. All interviews were conducted face-to-face in quiet, private consultation rooms at the hospital, scheduled at times convenient for participants, using a structured interview guide in simple Nepali to ensure clarity and cultural appropriateness.

#### Data analysis.

Data were analyzed using Colaizzi’s (1978) descriptive phenomenological method to explore the lived experiences of parents of children with autism in Nepal [9]. **Firstly**, all interviews were transcribed verbatim, translated into English from Neplai lamguage, and read repeatedly to immerse in the emotional, cultural, and social nuances of participants’ narratives. **Secondly**, significant statements reflecting emotional, physical, social, and financial experiences were extracted, with attention to culturally meaningful aspects such as family expectations and societal stigma. **Thirdly**, meanings were formulated while remaining faithful to participants’ perspectives. **Fourthly**, these meanings were organized into thematic clusters, including psychological stress, physical fatigue, social isolation, financial strain, and coping strategies. **Fifthly**, exhaustive descriptions were developed to integrate all themes into a comprehensive narrative of parents’ lived experiences. **Sixthly**, the fundamental structure of the phenomenon was identified, highlighting the balance of caregiving demands, emotional distress, resilience, and cultural values. **Finally**, findings were validated through member checking, confirming that the results accurately represented parents’ experiences within the Nepalese socio-cultural context.

Finally, in the Description phase, an integrated narrative synthesized these clusters into a coherent, contextually grounded account of parents’ lived experiences, from which the fundamental structure of the phenomenon was distilled. Member checking with a subset of participants confirmed the credibility and authenticity of the findings.

Throughout the analytic process, researchers engaged in rigorous bracketing to minimize personal bias, actively reflecting on their assumptions, cultural beliefs, and prior experiences related to autism and parenting in Nepal. This included suspending preconceptions about the causes of autism, caregiving roles within extended families, and societal stigma associated with developmental disabilities. To support this process, a reflexive journal was maintained, documenting analytic decisions, emerging insights, and reflections on how researchers’ positionality might influence interpretation. Peer debriefing with a qualitative research advisor provided critical oversight and helped challenge potential biases, while consultations with experts in autism and disability research ensured that interpretations were sensitive to the Nepalese socio-cultural and healthcare context. These experts offered guidance on culturally specific expressions, local idioms, and caregiving practices, such as the impact of *ijjat* (social reputation), community expectations, and reliance on informal social networks for support. Collectively, these strategies strengthened methodological rigor, credibility, and contextual validity, ensuring that findings authentically represented the lived experiences of parents raising autistic children in Nepal.

#### Trustworthiness.

To ensure trustworthiness, several practical strategies were applied throughout the study. The researcher maintained **bracketing and reflexive journaling** to set aside personal assumptions, which was especially important in the close-knit cultural context of Nepal where shared social beliefs could influence interpretation. **Credibility** was strengthened by reading and re-reading each transcript, cross-checking with audio recordings, and conducting **member checking** by revisiting participants in person or by phone to verify the accuracy of the interpreted meanings. **Dependability** and **confirmability** were supported through maintaining detailed field notes, documentation of analytic decisions, and regular consultation with an experienced **research expert** to review the coding and theme development process. **Transferability** was enhanced by providing thick descriptions of parents’ experiences and social settings, and by using **purposive sampling** to include parents from different socioeconomic and educational backgrounds, reflecting the diversity typical of Nepali families accessing public hospital services.

Throughout the analytic process, researchers engaged in rigorous bracketing to minimize personal bias, actively reflecting on their assumptions, cultural beliefs, and prior experiences related to autism and parenting in Nepal. This included suspending preconceptions about the causes of autism, caregiving roles within extended families, and societal stigma associated with developmental disabilities. To support this process, a reflexive journal was maintained, documenting analytic decisions, emerging insights, and reflections on how researchers’ presence might influence interpretation. Peer debriefing with a qualitative research advisor provided critical oversight and helped challenge potential biases, while consultations with experts in autism and disability research ensured that interpretations were sensitive to the Nepalese socio-cultural and healthcare context. These experts offered guidance on culturally specific expressions, local idioms, and caregiving practices, such as the impact of *ijjat* (social reputation), community expectations, and reliance on informal social networks for support. Collectively, these strategies strengthened methodological rigor, credibility, and contextual validity, ensuring that findings authentically represented the lived experiences of parents raising autistic children in Nepal.

### Trustworthiness

To ensure trustworthiness, several practical strategies were applied throughout the study. The researcher maintained bracketing and reflexive journaling to set aside personal assumptions, which was especially important in the close-knit cultural context of Nepal where shared social beliefs could influence interpretation. Credibility was strengthened by reading and re-reading each transcript, cross-checking with audio recordings, and conducting member checking by revisiting participants in person or by phone to verify the accuracy of the interpreted meanings. Dependability and confirmability were supported through maintaining detailed field notes, documentation of analytic decisions, and regular consultation with an experienced research advisor from PAHS to review the coding and theme development process. Transferability was enhanced by providing thick descriptions of parents’ experiences and social settings, and by using purposive sampling to include parents from different socioeconomic and educational backgrounds, reflecting the diversity typical of Nepali families accessing public hospital services.

Contextual Considerations: Nepal’s sociocultural context profoundly shapes parental experiences of ASD. Limited public awareness, social stigma, and scarcity of specialized services often intensify emotional and financial strain on families. The strong influence of collectivist values and extended family systems can both buffer and burden parents, affecting coping strategies and help-seeking behaviors. Conducting this study within such a context provided an opportunity to illuminate the intersection of cultural beliefs, healthcare accessibility, and caregiving practices areas that have been largely overlooked in South Asian autism research.

#### Adequacy and data saturation.

In phenomenological research, data saturation is reached when no new themes or insights emerge from interviews rather than achieving numerical completeness. In this study, saturation was monitored iteratively through concurrent data collection and analysis. By the sixth interview, recurring themes such as emotional burden, social stigma, financial strain, and adaptive coping were identified, and by the seventh, data became repetitive. The eighth interview confirmed thematic stability, and the ninth served as a confirmatory case. Saturation was determined jointly by the researcher and advisor, ensuring all major themes were well-supported. The nine participants provided rich, information-dense data sufficient to address the study’s aims.

## Results and discussion

Table 1 depicits that mean age of eight mothers: **36.33 years**. One of respondent was father.(SD: **6.35**).More than half of respondents, i.e., 55% belonged to the **Bhramin** ethnic group.

**Table 1:** Sociodemographic information of parents directly involved in child care, N = 9.

Respondent	Age	Religion	Ethnicity	Education	Occupation	Monthly Family Income (NPR)
1. Mother	39	Hindu	Bhramin	12 class	Business	2 Lakhs
2. Mother	27	Hindu	Chhetri	8 Class	Housewife	50K
3. Mother	36	Christian	Janajati	Bachelor	Foreign/Caretaker	8-10 Lakhs
4. Mother	38	Hindu	Bhramin	12 class	Housewife	1 Lakh
5. Father	51	Christian	Bhramin	10 class	Service	85K
6. Mother	33	Hindu	Bhramin	12 class	Homemaker	2 Lakhs
7. Mother	35	Hindu	Janajati	12 class	Homemaker	30K
8. Mother	38	Hindu	Janajati	Bachelor	Business	1 Lakh
9. Mother	27	Hindu	Bhramin	Bachelor	Homemaker	50K

Table 2 depicts that Mean age of diagnosis of ASD was 2.22 years with SD 0.634.Majority of child were male whereas only one was female. More than half of affected child, i.e., 55.55% has sibling.

**Table 2:** Sociodemographic information of the children, n = 10.

Child	Age at Diagnosis	Present Age	Sex	Continuity of Care	Sibling
1	2 years	5 years	Male	Yes	1
2	2 years	4 years	Male	Yes	–
3	18 months	6 years	Male	Yes	1
4	3 years	9 years	Male	Yes	2
5	3 years	7 years	Male	Yes	1
6	3 years	6 years	Male	Yes	–
7	2.5 years	8 years	Male	Yes	–
8	2 years	7 years	Male, Male (Twins)	Yes	Twins
9	3 years	7 years	Female	Yes	–

Table 3 summarizes the themes and sub-themes from interviews with nine parents of children with autism in Nepal. Parents reported emotional turmoil, physical exhaustion, social isolation, financial strain, and disrupted livelihoods, yet many demonstrated resilience and deep commitment to their children. Psychological impact, especially among mothers, included guilt, regret, and fluctuating hope regarding their child’s progress. Coping strategies involved hopeful thinking, focusing on therapies, and support from extended family, though feelings of isolation often persisted.

**Table 3:** Emergent themes and sub-themes (N = 9).

Theme	Sub-Theme
**Psychological Impact**	Emotional Burden
	Hope & Uncertainty
	Regret & Guilt
**Physical Impact**	Fatigue & Strain
	Sleep Deprivation
	Child Safety
**Financial Impact**	High Cost of Treatment
	Limited Resources
	Sibling Impact
**Social Impact**	Social Isolation & Embarrassment
	Family Sacrifices
**Career Impact**	Job Loss & Career Sacrifices
	Employment Challenges

**The supporting Verbatim Responses** are as follows

Psychological Impact:

*“I am solely the mental sufferer for this child. His father lives abroad. He does not take as much stress as I do.”* P-1

*“I am stressed mentally since I am busy taking care of his younger brother as well. However, his grandparents used to take care of him. Sometimes I feel diverted as well”.* P-4

*“We regret giving birth to this child because we already had a normal child before. Because of this special child, the childhood (Balapaan) of a normal child is also disturbed.”*P-5

*“The mental stress is unexplainable. We, husband and wife, are in different countries due to the child’s condition. I have to return to Nepal.”*P-6

The **Physical Impact** theme highlighted the immense physical toll caregiving demands place on parents. Many described ongoing **fatigue**, **sleep deprivation**, and concerns regarding their child’s **physical safety** due to unpredictable and sometimes dangerous behaviors. These challenges often persisted over the years, with increasing intensity as the child grew older and physically stronger.

**Theme**: Fatigue, Sleep Deprivation, Managing Physical Safety

**Physical Rest and Routine**:

To mitigate the fatigue, some parents establish structured routines, though they often feel physically exhausted. Parents take extra precautions and engage in preventive strategies to minimize the risk of injury.

The supporting Verbatim Responses are as follows

*“The child is getting bigger, and he pulls us; he hits us. We feel tired from caring for this child throughout the day. We have to do every routine work”* P-4

*“The child used to sleep during the day and cried throughout the night. Because of this, I was sleepy throughout the day at my job*.” P-5

*“I am only the caretaker of this child. Along with household chores, I used to get tired a lot.”* P-8

**Social Impact** emerged as another significant domain, with parents frequently reporting experiences of **social isolation**, **embarrassment**, and **emotional pain** due to stigmatizing societal attitudes. Avoidance of social functions was common, and some families reported making sacrifices, such as staying home during events, to prevent judgment or distress.

**Theme**: Social Isolation, Embarrassment, Family Sacrifices

**Avoidance of Negative Social Interactions**: Parents tend to limit their social engagements to avoid stigmatizing encounters.

**Internal Resilience and Defensiveness**: Some parents use deflection techniques to shield their children from negative societal judgments.

The supporting Verbatim Responses are as follows

*“It is very embarrassing to take the child to social functions. People say many offensive things such as dumb child. Nowadays, I am limiting my social life.”* P-4

*“Her eyes were tearful. Tears rolled on her cheeks. She said, ‘I don’t like to interact with other people in any function due to this child.”* P-9

“*We held back from our social life and functions for almost 3 years. People used to say better not to bring such a child to social functions like marriage ceremonies. It hurts sometimes.”* P-6

*“I only returned to Nepal along with my child. Everyone in society is concerned about my return, so I stopped going to such places.”* P-7

Under **Impact on Career**, several parents, particularly mothers, had either reduced their working hours or quit employment entirely to provide full-time care for their child. Others shared difficulties related to **immigration or Permanent Residency eligibility** in foreign countries because of their child’s diagnosis. Limited employment flexibility and reduced career advancement opportunities further contributed to family stress.

**Theme**: Job Loss, Career Sacrifices, Employment Challenges

**Career Adjustments**: Parents make career sacrifices, often choosing part-time work or quitting jobs to prioritize caregiving.

**Adaptation to Changing Employment Landscape**: Some parents continue working but adjust their career expectations in response to caregiving needs.The supporting Verbatim Responses are as follows

*“We are having a negative effect on our career. We hear that, owing to this type of child, we will not be permitted PR in the foreign country where we are working.” P-3*

*“We two family members (me and my mother-in-law) quitted our jobs to take care of this child.”* P-4

*“I used to work full-time and part-time before the diagnosis. However, now, I work only at one place.”* P-5

The theme of **Financial Impact** illustrated the economic strain that families endure due to the high cost of special education, therapy, and treatment. Participants repeatedly emphasized how limited income and the need for specialized services placed a burden on the family as a whole. In some cases, the needs of neurotypical siblings were also compromised due to redirected resources.

**Theme**: High Cost of Treatment, Limited Financial Resources

**Financial Prioritization**: Parents make difficult financial decisions, often prioritizing their child’s care over other expenses.

**Seeking Alternative Support**: Some parents actively seek financial assistance or community support to manage the financial burden. The supporting Verbatim Responses are as follows

*“We don’t have any source of income. I am not sure that I could continue his treatment. Schools do not like to enroll the child. Special schools are costly.”*P-2

*“It is very difficult to manage for treatment and therapies. He dropped out of school, saying they could not handle this child. One special school demanded NPR 40,000 a month along with therapies.”* P-4

*“It is very difficult to invest 60-70% of my salary in special schools. There are other things, like play materials, we should afford*” P-5

*“I used to earn a lot. Since I had to come back home, I lost every earning opportunity.”* P-6

### Coping strategies used by parents

#### Psychological coping strategies.

Parents employed various coping mechanisms to manage emotional stress, including seeking support from extended family and engaging in religious or hopeful thinking. Some parents tried to emotionally accept their child’s condition and continued believing in the potential progress through therapy. **The supporting Verbatim Responses are as follows:**

*“I am stressed mentally since I am busy taking care of his younger brother as well. However, his grandparents used to take care of him.”* ***P-4***

*“I try to divert my mind by listening to religious Bhajans.”* ***P-1***

*“We believe that therapy could help him improve.”* ***P-9***

*“We are trying to accept the child’s condition.”* ***P-2***

#### Physical coping strategies.

Parents attempted to maintain structured caregiving routines to manage fatigue and ensure safety. Although exhaustion persisted, they continued to adjust their daily schedules and caregiving approaches. **The supporting Verbatim Responses are as follows:**

*“The child is getting bigger, and he pulls us; he hits us. We feel tired from caring for this child throughout the day. We have to do every routine work.”* ***P-4***

*“The child used to sleep during the day and cried throughout the night. Because of this, I was sleepy throughout the day at my job.”* ***P-5***

#### Social coping strategies.

Parents coped by limiting participation in social events to avoid stigma. Some developed emotional resilience by ignoring negative comments and prioritizing the child’s emotional comfort. **The supporting Verbatim Responses are as follows:**

*“It is very embarrassing to take the child to social functions. People say many offensive things such as dumb child. Nowadays, I am limiting my social life.”* ***P-4***

*“Her eyes were tearful. She said, ‘I don’t like to interact with other people in any function due to this child.’”* ***P-9***

*“We held back from our social life and functions for almost 3 years.”* ***P-6***.

*“I stopped going to such places.”* ***P-7***

#### Career-related coping strategies.

To cope with caregiving responsibilities, parents made career adjustments such as reducing work hours or quitting jobs. Some reassessed career expectations to manage caregiving demands. **The supporting Verbatim Responses are as follows:**

*“We two family members (me and my mother-in-law) quit our jobs to take care of this child.”* ***P-4***

*“I used to work full-time and part-time before the diagnosis. However, now, I work only at one place.”* ***P-5***

#### Financial coping strategies.

Families prioritized treatment expenses, sought support where possible, and reduced non-essential spending to sustain care. Some actively pursued government or community assistance. **The supporting Verbatim Responses are as follows:**

*“We don’t have any source of income. I am not sure that I could continue his treatment. Schools do not like to enroll the child. Special schools are costly.”* ***P-2***

*“It is very difficult to manage for treatment and therapies. He dropped out of school, saying they could not handle this child.”* ***P-4***

*“It is very difficult to invest 60–70% of my salary in special schools. There are other things, like play materials, we should afford.”* ***P-5***

## Discussion

The study explored the lived experiences of nine mothers raising children with autism through in-depth interviews, highlighting significant physical, social, and emotional impacts. Mothers reported feelings of guilt, regret, loneliness, frustration, non-acceptance, hopelessness, and depression, reflecting high psychological burden. These findings align with recent Asian studies, where caregiver burden has been linked to children’s behavioral challenges, social stigma, and limited support systems. For example A study done in 2023 reported elevated caregiver stress among parents of children with autism in Malaysia [10], while Fithriyah and Carrasco [11] found psychosocial distress and anxiety in Indonesian mothers. Similarly, another study highlighted the impact of caregiving on occupational balance and emotional well-being among parents in Malaysia [12]. Across these contexts, the compounded effects of social isolation, financial strain, and limited institutional support exacerbate maternal emotional distress, underscoring the influence of cultural and systemic factors on parental experiences in Asia.

### Physical impacts

One of the most prevalent concerns expressed by parents in this study was the **chronic lack of sleep and persistent tiredness** resulting from their child’s irregular sleeping patterns, frequent night awakenings, and heightened sensory sensitivities. This continuous exhaustion affected their overall well-being, reducing energy levels, concentration, and emotional stability, while increasing psychological distress. Similar findings have been widely documented in recent literature, showing that sleep disturbances in children with autism have a direct and significant impact on parental health and functioning [13,14].

Recent studies have shown a bidirectional relationship between children’s sleep problems and parental stress: sleep disturbances in children increase caregiver fatigue, while high parental stress can, in turn, worsen children’s sleep patterns [13,15]. This cycle places continuous strain on the family and underscores the need for early interventions that address both child and parent sleep issues.

In low- and middle-income countries (LMICs) like **Nepal**, the consequences of this pattern are intensified by limited access to specialized services, high out-of-pocket costs, and the absence of respite care. Sleep deprivation thus becomes intertwined with broader socioeconomic hardship, particularly for mothers who are often the primary caregivers [16]. Similar LMIC-based research has emphasized that parents’ lack of sleep contributes to reduced work productivity, strained marital relationships, and diminished quality of life [13,15].

Recent intervention studies suggest that parent-mediated sleep interventions including structured bedtime routines, behavioral sleep strategies, and psychoeducation on sleep hygiene can significantly improve both child and parent sleep quality [17]. Implementing such evidence-based, low-cost approaches in Nepal could help mitigate parental fatigue and promote family well-being, especially when delivered through community-based or task-sharing models that align with local cultural and resource constraints.

### Social impacts

Social isolation emerged as another prominent theme. Parents reported feeling disconnected from their extended families and communities, largely due to the demanding nature of caregiving responsibilities. Many parents expressed that their social interactions were limited, as they often felt misunderstood by others or faced stigma associated with their child’s condition. Their social lives were significantly affected, as they were unable to participate in gatherings, celebrations such as marriages and Bratabandha (a coming-of-age ceremony), and other communal activities. The lack of social support and understanding from society further deepened their sense of isolation, aligning with findings from previous research that highlighted the social withdrawal experienced by parents of children with autism [18]. Similarly, a study by Pottie and Ingram [19] revealed that parents of children with autism reported lower levels of social support and higher levels of perceived stress than other parents did. The same study by Wang [20] noted that parents of children with ASD had less social support, leading to increased feelings of isolation.

These findings are consistent with a growing body of international evidence indicating that parents of children with autism often experience significant social marginalization and reduced perceived social support. \ A study done by Wang et al. [20] found that lower levels of social support were strongly associated with increased feelings of loneliness and psychological distress among parents of children with ASD. Similarly, recent research in Asia and LMIC settings has highlighted that cultural stigma, community misconceptions about autism, and lack of institutional inclusion contribute substantially to parental social withdrawal and emotional strain [21,22].

Studies from neighboring countries provide further parallels. In India and Bangladesh, researchers have documented that caregivers often avoid community events and limit social engagement to escape judgment or pity, resulting in emotional exhaustion and reduced access to informal support networks [22,23]. Comparable findings were also reported in Nepal, where families of children with developmental disabilities face negative labeling, social exclusion, and limited understanding from peers and relatives [24]. These studies collectively underscore how sociocultural stigma, lack of awareness, and insufficient community support reinforce parents’ sense of isolation and distress.

In this context, parents in Nepal demonstrate remarkable adaptive coping strategies, such as relying on close family members, faith-based communities, and self-isolation as protective measures to minimize distressing encounters. However, while such strategies may offer temporary relief, they also risk perpetuating further social withdrawal and emotional fatigue. Therefore, increasing public awareness, inclusive education, and community-based support programs remains essential to mitigate social isolation and enhance the social well-being of families raising children with autism in Nepal.

### Educational impact

Another critical finding was the difficulty in enrolling children with autism in regular schools. Many parents reported facing rejection from mainstream educational institutions, which often cited a lack of resources or expertise to accommodate children with special needs. This exclusion placed additional stress on parents, who struggled to find appropriate educational opportunities for their child. The challenge of securing proper schooling options further reinforced parents’ sense of helplessness and exclusion from mainstream societal functions, echoing existing literature that emphasizes the barriers to inclusive education for children with autism [25].

Recent studies corroborate these barriers to inclusive education for children with autism. For example, a 2024 study of preschool teachers in Ghana found that significant obstacles were inadequate teacher training, limited parental cooperation, and societal stigma, all of which hinder the inclusion of children with autism in mainstream classrooms [26]. Similarly, research in Nepal highlights how lack of trained personnel, rigid curricula, and poor infrastructure continue to limit access for children with autism to inclusive educational settings [16].

Together, these findings suggest that beyond individual family challenges, structural, institutional and sociocultural barriers persist that restrict children with autism from meaningful inclusion in mainstream education. In the context of Nepal, where policy frameworks such as the Disability Rights Act and inclusive-education guidelines are relatively recent and resources remain limited, parents’ experiences of exclusion reflect both micro-level (school refusal, resource inadequacy) and macro-level (policy implementation, societal attitudes) obstacles.

Given this, it is imperative that inclusive education efforts not only focus on placing children with autism in mainstream classrooms, but also address teacher training, resource allocation, adapted curricula, and community awareness. A concerted approach is needed to ensure that the right to education becomes a reality rather than a nominal policy statement.

### Professional impacts

Parents faced challenges in managing their professional lives, with many struggling to balance work and caregiving responsibilities. Some reported losing their jobs due to time constraints, whereas others had to return to Nepal from foreign employment to take care of their child. The difficulty in time management further compounded their stress, making it challenging to maintain a stable career.

Parents in this study often described significant disruptions to their professional lives as a result of caregiving responsibilities for a child with autism. Many reported having to reduce working hours, quit their jobs, or relocate (e.g., returning to Nepal from abroad) to provide full-time care. These decisions compounded the stress of balancing caregiving and work, undermining their career stability and financial security.

Recent quantitative and qualitative research supports these findings. A large cross-sectional study of 5,018 Chinese families found that only 37.3% of mothers of children with autism spectrum disorder (ASD) remained employed, compared to 96.7% of fathers; moreover, 54.3% of mothers had resigned from employment to care for their children, while only 2.8% of fathers had done so [27]. Similarly, results from the U.S.-based R-Kids study indicated that parents of children with ASD were significantly less likely to be employed than parents of children with asthma or no chronic condition and experienced substantially greater productivity losses at work [28].

In the Nepali context, the qualitative accounts from parents returning from employment abroad reflect both global patterns and local particularities: migration for work is common, and caregiving demands in the absence of sufficient support systems mean that parents may choose to end foreign employment or reduce professional engagement, thereby compromising both income and career progression. These professional sacrifices add a layer of financial and emotional burden, intersecting with the themes of financial strain and parental resilience already identified.

Given these impacts, interventions and policy responses should consider flexible working arrangements, employer-awareness of caregiving burdens, cross-sectoral support for families (especially those with diaspora or migrant employment histories), and career counselling or reintegration programs for parents whose work has been interrupted. Ensuring parents can remain engaged in meaningful work perhaps part-time or remote with flexibility may reduce stress and protect family income while recognizing the caregiving reality.

### Economic impacts

Raising a child with autism placed a significant financial burden on families. Parents reported high expenses related to therapies, special education, and costly childcare centers. Many also faced economic strain due to job loss, further exacerbating their financial difficulties. The high cost of specialized services made it difficult for some families to access adequate care, highlighting the need for financial support programs for these parents.

Recent international research supports these findings. Large-scale studies have documented substantial direct and indirect costs for families of children with autism spectrum disorder (ASD), including therapy, medical services, educational expenses, and lost income due to reduced employment [29,30].

In the Nepalese context, recent studies corroborate these challenges. A study done in 2025 found that parents of children with ASD attending a tertiary hospital in Kathmandu experienced moderate to severe burden, with financial strain as a major contributor [31]. Another study done in Nepal reported that high out-of-pocket costs for therapies and specialized educational services, coupled with limited community and institutional support, exacerbated economic pressures for Nepalese families [32]. Similarly, Shrestha and Shrestha (2023) found that financial stress was a significant component of caregiver burden in central Nepal [33], and Koirala and Khatri highlighted that families often faced economic hardship and social exclusion due to expenses associated with care and education [32].

These findings suggest that, beyond the individual household level, structural barriers such as limited government subsidies, lack of affordable special education, and inadequate community support compound economic stress in Nepal. Policy interventions, including financial assistance for therapies, subsidies for special education, income support for caregivers, and affordable community-based services, are urgently needed to reduce the financial burden on families and improve access to appropriate care.

### Sibling impacts

The presence of a child with autism in the family also affected their siblings. Many parents expressed concerns about the loss of childhood experiences for their other children, as their primary focus remained on the child with autism. Some siblings experienced irritation and received less attention, leading to feelings of neglect and frustration.

Recent studies have further explored the developmental and social dynamics experienced by siblings of children with autism spectrum disorder (ASD). A 2024 study by UC Davis Health found that siblings of children with ASD had a 20% likelihood of receiving an autism diagnosis themselves approximately seven times higher than the rate observed among infants without autistic siblings [34]. Similarly, research published in *Frontiers in Psychiatry* indicated that siblings of children with autism often exhibit poorer peer relationships, reduced peer involvement, and lower academic functioning compared to their peers [35]. Nonetheless, positive outcomes have also been documented, with these siblings demonstrating enhanced prosocial behaviors, stronger social support networks, and greater perspective-taking abilities [35].

### Interpersonal relationship impacts

Caregiving responsibilities often placed a greater burden on mothers, who took on the primary role in caring for their child. However, some parents highlighted positive aspects, such as the family uniting to support the child and spending more time together. Despite the stress, the shared responsibility in some families strengthened family bonds, fostering a sense of teamwork and understanding.

Recent evidence highlights the importance of intimate parental relationships in coping with complex caregiving demands. In one study of 201 married couples raising a school-aged child with autism spectrum disorder (ASD), higher levels of parental intimacy were positively correlated with each parent’s own dyadic coping even though self-disclosure did not show a direct correlation. Notably, the partner effect of fathers’ intimacy on mothers’ dyadic coping was statistically significant, underscoring the interdependent nature of coping processes in these families [36].

### Coping strategies

Parents adopt various coping mechanisms to manage their emotional stress. Common strategies include crying, listening to religious music (Bhajans), meditating, and practicing positive thinking. Some parents find solace in viewing their experiences as an opportunity to educate and support other parents facing similar challenges.

A 2025 study revealed that 100% of parents of children with autism spectrum disorder (ASD) and attention-deficit/hyperactivity disorder (ADHD) reported utilizing emotion-focused coping strategies, whereas 94.93% of parents of children with neurotypical development primarily employed problem-focused coping strategies [35]. Similarly, another study demonstrated that interventions such as Acceptance and Commitment Therapy (ACT), Cognitive Behavioral Therapy (CBT), and psychoeducation significantly reduce stress, anxiety, and depression among parents of children with autism [36].

Parents of children with autism in Nepal employed multiple strategies to manage the challenges of caregiving. Emotionally, they practiced self-regulation, acceptance, and maintained hope through therapy progress. Many relied on spiritual practices, including listening to religious songs or bhajans, prayer, and faith-based reflection, which helped them find comfort and inner strength. Support from spouses, extended family, and friends provided practical assistance and emotional reassurance. To cope with physical strain and sleep disruption, parents established structured routines, rest periods, and constant supervision to ensure child safety. Career and financial pressures were managed by adjusting work schedules, prioritizing expenses, and seeking community or government assistance. Parents also balanced the needs of siblings by carefully allocating resources and fostering family cohesion. Across these strategies, parents demonstrated resilience, adaptability, and patience, enabling them to sustain care and support their child’s development despite social stigma and limited institutional resources.

#### Suggestions for improvement.

To alleviate the significant burdens faced by parents of children with autism spectrum disorder (ASD), several key recommendations have emerged from recent studies. First, ensuring accessible education is essential; government schools should establish dedicated programs for children with special needs to promote inclusion and equal learning opportunities. Second, the high fees of private autism care centers should be reduced to make therapeutic and educational services more affordable for families. Third, coordination between therapists and schools must be strengthened to improve communication, consistency, and the integration of care across settings. Fourth, teachers should receive specialized training to effectively support the learning and behavioral needs of children with autism. Finally, the establishment of parental support groups is recommended to provide emotional support and a platform for parents to share experiences, coping strategies, and resources. Collectively, these measures aim to reduce parental stress and enhance the overall well-being of families affected by autism.

The need for systemic support is echoed in the literature. Wang [20] highlighted parents’ need for better health services, strengthened home support systems, and more increased state support during challenging time [20].

## Conclusion

This study highlights the multifaceted challenges faced by parents of children with Autism Spectrum Disorder (ASD) in Nepal, including psychological, physical, social, career, and financial burdens. Parents employ coping strategies such as emotional regulation, seeking support, and financial sacrifices, yet challenges remain substantial. The findings underscore the need for greater social acceptance, improved access to special education and therapies, and financial support programs. Enhanced government involvement and inclusive societal attitudes are essential to better support families, improve quality of life, and promote the well-being of both parents and children.

## Supporting information

S1 File Detailed qualitative descriptions of parents’ experiences and the coping strategies they used. This material includes illustrative examples and expanded explanations that support and contextualize the findings reported in the main manuscript.
(DOCX)
