# Supplementary material for: Experience and coping strategies of parents of children with autism: A qualitative study
Source: PLoS One. 2025 Dec 23;20(12):e0339349. doi: 10.1371/journal.pone.0339349 (PMC12725599; doi:10.1371/journal.pone.0339349)
Supplement: S1 File — This material includes illustrative examples and expanded explanations that support and contextualize the findings reported in the main manuscript. (DOCX) [file pone.0339349.s001.docx]

**Coping Strategies used by parents having child with ASD, N=9**

| **Theme** | **Sub-Theme** | **Coping Strategies** | **Example from Verbatim Responses** |
| --- | --- | --- | --- |
| **Psychological Impact** | Emotional Burden  Hope and Uncertainty | Emotional Regulation: Parents attempt to manage mental stress through hopeful thinking and focus on therapeutic interventions. Focusing on listening religious song Bhajans for diversion. | “I am solely the mental sufferer for this child. His father lives abroad. He does not take much stress as I do.” (Respondent 1) |
|  |  | Social Support: Some parents lean on extended family to share caregiving responsibilities. | “I am stressed mentally since I am busy taking care of his younger brother as well. However, his grandparents used to take care of him.” (Respondent 4) |
|  |  | Focus on Improvement: Parents use hopeful thinking and believe therapy could lead to progress. | “We regret giving birth to this child because we already had a normal child before. Because of this special child, the childhood (Balapaan) of a normal child is also disturbed.” (Respondent 5) |
|  |  | Accepting Child’s Condition: Parents reflect on emotional challenges but try to accept their child’s condition. | “The mental stress is unexplainable. We husband and wife are in different countries due to the child’s condition. I have to return back to Nepal.” (Respondent 6) |
| **Physical Impact** | Fatigue and Physical Strain | Physical Rest and Routine: Some parents maintain routines, although physical exhaustion persists. | “The child is getting bigger and he pulls us; he hits us. We feel tired from caring for this child throughout the day.” (Respondent 4) |
|  |  | Caregiving Routines: Parents manage physical demands through established routines but continue to experience fatigue. | “The child used to sleep during the day and cried throughout the night. Because of this, I was sleepy throughout the day at my job.” (Respondent 5) |
| **Social Impact** | Social Isolation | Avoidance of Negative Social Interactions: Parents limit social engagements to avoid judgment and exclusion. | “It is very embarrassing to take the child to social functions. People say many offensive things such as dumb child.” (Respondent 4) |
|  |  | Internal Resilience: Some parents actively defend their child from negative social comments and seek to minimize the impact of social stigma. | “Her eyes were tearful. She said, 'I don’t like to interact with other people in any function due to this child.'” (Respondent 9) |
|  |  | Social Withdrawal: Parents withdraw from social functions to protect their child from stigmatization. | “We held back from our social life and functions for almost 3 years. People used to say better not to bring such a child to social functions like marriage ceremonies.” (Respondent 6) |
| **Impact on Careers** | Job Loss and Career Sacrifices | Career Adjustments: Parents make sacrifices in their careers by reducing work hours or quitting jobs to prioritize caregiving. | “Two family members had to quit their jobs to take care of this child.” (Respondent 4) |
|  |  | Adaptation to Changing Employment Landscape: Some parents continue to work, but they adjust career expectations to accommodate caregiving needs. | “I used to work full-time and part-time before the diagnosis. However, now, I work only at one place.” (Respondent 5) |
| **Financial Impact** | High Cost of Treatment | Financial Prioritization: Parents prioritize caregiving costs over other household expenses, even sacrificing essential needs. | “We don’t have any source of income. I am not sure that I could continue his treatment. Schools do not like to enroll the child. Special schools are costly.” (Respondent 2) |
|  |  | Seeking Alternative Support: Parents seek financial assistance from government programs or community support. | “It is very difficult to manage for treatment and therapies. He dropped out of school, saying they could not handle this child.” (Respondent 4) |
|  |  | Budgeting for Special Needs: Parents manage expenses by reducing discretionary spending and focusing on what is necessary for the child’s needs. | “It is very difficult to invest 60-70% of my salary in special schools. There are other things, like play materials, we should afford.” (Respondent 5) |

**Emergent Themes, Sub-Themes, and Illustrative Quotes from Parent Interviews (N=9)**

| **Theme Category** | **Sub-Themes** | **Description** | **Example from Verbatim Responses** |
| --- | --- | --- | --- |
| **Psychological Impact** | Emotional Burden on Mothers | Mothers feel primarily responsible for caregiving, leading to extreme mental stress. Fathers abroad experience comparatively less stress. | “I am solely the mental sufferer for this child. His father lives abroad. He does not take much stress as I do.” |
|  | Hope and Uncertainty | Parents experience fluctuating emotions—sometimes hopeful about improvements through therapy but also facing despair. | “Sometimes I am stressed, but sometimes I think that he will be normal through these therapies and treatment.” |
|  | Regret and Guilt | Some parents express regret about having a child with ASD, especially due to its impact on their other children. | “We regret giving birth to this child because we already had a normal child before.” |
|  | Sibling Neglect and Emotional Strain | Parents feel guilty that the neurotypical siblings experience a loss of childhood and reduced parental attention. | “His elder sister had suffered a lot because of more attention, love, and care to this child with ASD.” |
| **Physical Impact**  **Social Impact** | Fatigue and Physical Strain | Parents feel exhausted due to the high-energy demands of their child and their continuous care responsibilities. | “The child is getting bigger, and he pulls us, he hits us. We feel tired caring for this child throughout the day.” |
|  | Sleep Deprivation | Parents suffer from extreme exhaustion due to irregular sleep patterns of the child. | “The child used to sleep during the day and cried the whole night. Because of this, I was sleepy throughout the day at my job.” |
|  | Managing Physical Safety | Parents are constantly worried about their child’s safety due to risky behaviors. | “Once he climbed a chair and tried to stop the running ceiling fan. His head was injured as well.” |
|  | Social Isolation and Embarrassment | Parents avoid social functions due to negative societal attitudes and offensive remarks. | “It is very embarrassing to take the child to social functions. People say many offensive things like ‘dumb child.’” |
|  | Emotional Distress in Social Settings | Parents feel unwelcomed and emotionally distressed in social gatherings. | “Her eyes were tearful. She said, ‘I don’t like to interact with other people in any function due to this child.’” |
|  | Family Sacrifices for Social Inclusion | Some family members stay home to care for the child instead of attending social events. | “His grandparents used to stay with him at home instead of attending any functions.” |
| **Impact on Career** | Job Loss and Career Sacrifices | Parents, especially mothers, have to quit their jobs to provide full-time care. | “Two family members had quit their jobs to take care of this child.” |
|  | Foreign Employment and PR Challenges | Parents struggle with visa or PR issues in foreign countries due to their child’s ASD. | “We heard that due to this type of child, we will not be permitted PR in a foreign country where we are working.” |
|  | Limited Work Opportunities | Even when employed, parents face restrictions on career growth due to caregiving responsibilities. | “I used to work full-time and part-time before the diagnosis. However, now, I work only at one place.” |
| **Financial Impact** | High Cost of Special Education and Therapy | Parents struggle to afford private special schools and therapies. | “One of the special schools demanded NPR 40K a month along with therapies and education.” |
|  | Limited Financial Resources | Families without stable income sources find it difficult to continue their child’s treatment and education. | “We don’t have any source of income. I am not sure that I could continue his treatment.” |
|  | Financial Sacrifices Affecting Siblings | Other children face limitations in basic needs due to the financial burden of ASD-related expenses. | “Even his sister is having limitations in essential stuff due to expenditure on this child.” |
| **Suggestions** | Government-Supported Special Education and Therapy Centers | Parents urge the government to establish affordable special schools with integrated therapies. | “Government should open special education schools along with therapies for such children.” |
|  | Coordination Between Medical and Educational Sectors | Improved collaboration between medical professionals and educators is necessary. | “Government should coordinate with medical personnel on this.” |
|  | Financial Assistance for Families | Parents demand financial support programs to alleviate the high cost of treatment and education. | “General Nepali people can’t afford private schools for special education.” |
